# Supplementary material for: Terahertz-field activation of polar skyrons
Source: Nat Commun. 2025 Oct 9;16:8994. doi: 10.1038/s41467-025-64033-6 (PMC12511383; doi:10.1038/s41467-025-64033-6)
Supplement: Supplementary file 1 — Supplementary Information [file 41467_2025_64033_MOESM1_ESM.pdf]

Supplementary Information for

Terahertz-field activation of polar skyrons

Huaiyu (Hugo) Wang[1,2]†, Vladimir A. Stoica[1,3]†, Cheng Dai[1]†, Marek Paściak[4]†, Sujit Das[5], Tiannan Yang[1,17], Mauro A. P. Gonçalves[4], Jiri Kulda[6], Margaret R. McCarter[7], Anudeep Mangu[8], Yue Cao[9], Hari Padma[1],Utkarsh Saha[1], Diling Zhu[10], Takahiro Sato[10], Sanghoon Song[11], Matthias C. Hoffmann[11], Patrick Kramer[11], Silke Nelson[11], Yanwen Sun[11], Quynh Nguyen[11], Zhan Zhang[3], Ramamoorthy Ramesh[7,11,12,13,14,15], Lane W. Martin[11,13,14,15,16], Aaron Lindenberg[2,8], Long-Qing Chen[1], John W. Freeland[3]*, Jirka Hlinka[4]*, Venkatraman Gopalan[1]*, Haidan Wen[3,9]*

[1]Department of Materials Science and Engineering, The Pennsylvania State University; University Park, PA, USA.

[2]Stanford Institute for Materials and Energy Sciences, SLAC National Accelerator Laboratory; Menlo Park, CA, USA.

[3]Advanced Photon Source, Argonne National Laboratory; Lemont, IL, USA.

[4]Institute of Physics of the Czech Academy of Sciences; Prague, Czech Republic.

[5]Materials Research Centre, Indian Institute of Science, Bangalore, India

[6]Institut Laue Langevin; 71 avenue des Martyrs, 38000 Grenoble, France.

[7]Department of Materials Science and Engineering, University of California, Berkeley; Berkeley, CA, USA.

[8]Department of Materials Science and Engineering, Stanford University; Stanford, CA, USA.

[9]Materials Science Division, Argonne National Laboratory; Lemont, IL, USA.

[10]Linac Coherent Light Source, SLAC National Accelerator Laboratory; Menlo Park, CA, USA.

[11]Materials Sciences Division, Lawrence Berkeley National Laboratory; Berkeley, CA, USA.

[12]Department of Physics, University of California, Berkeley; Berkeley, CA, USA.

[13]Department of Materials Science and NanoEngineering, Rice University; Houston, TX, USA.

[14]Department of Physics and Astronomy, Rice University; Houston, TX, USA.

[15]Rice Advanced Materials Institute, Rice University; Houston, TX, USA.

[16]Department of Chemistry, Rice University; Houston, TX, USA.

[17]Present address: Interdisciplinary Research Center, School of Mechanical Engineering, Shanghai Jiao Tong University, Shanghai, China.

* Corresponding authors. Email: freeland@anl.gov, hlinka@fzu.cz, vxg8@psu.edu, wen@anl.gov

†These authors contributed equally to this work.

Supplementary Text 1: Analytical approximation of dynamical diffraction intensity changes in polar skyrmions.
Supplementary Note 2: Calibration between computational and experimental results.

Table S1. Parameters for dynamic phase field simulations and diffraction pattern calculation.

Figure S1. Dynamics of polar skyrmion and vortices near 004 Bragg peak.
Figure S2. Dynamical phase-field simulation of THz-driven polar skyrmions and polar vortices.
Fig. S3. Simulations of the static and dynamic diffraction intensity from skyrmion domains and domain walls.
Fig. S4. Phonon dispersion calculated by the atomistic model and compared with experimental results.
Fig. S5. Polarization changes at the peak of their motions calculated by the atomistic model.
Fig. S6. Pb displacements at the peak of their motions calculated by the atomistic model.
Fig. S7. Shear strain induced by mode A and B.
Fig. S8. Temperature and field dependence of the dynamical and static responses in polar skyrmions.
Fig. S9. Dynamical response of the polar skyrmions to 400 nm optical pump at 300 K.
Fig. S10. Calibrate atomistic results to experimental observations.

Supplementary Text 1: Analytical approximation of dynamical diffraction intensity changes in polar skyrmions

In this session, we employ an analytical approximation, using phase-field results as inputs, to explain the out-of-phase dynamical signal observed between the m = +1 and m = -1 satellite peaks. Additionally, we will use the same approach to clarify why, in a polar vortex, the dynamical signal between these satellite peaks remains in phase.

The phase-field simulation first found the ground state in which the polar skyrmion and polar vortex structures are stabilized. Then, the polarization modulation at a time delay corresponding to the local maximum/minimum of the intensity change at the satellite peaks were calculated following the established method[1] (Fig. S2). In this study, we adopt a simplified approach to gain physical insights into the simulation result. Specifically, we compute the structure factor of polar skyrmion bubble as follows:

$$F(\mathbf{Q}) = \sum_n f_{pb} e^{(-i\mathbf{Q}\cdot\mathbf{R}_{Pb,n})} + f_{Ti} e^{(-i\mathbf{Q}\cdot\mathbf{R}_{Ti,n})} \quad \text{(S1)}$$

The satellite peaks are close to the Bragg lattice peak 004 and our calculation is based on this Bragg peak choice. $\mathbf{Q} = (\pm\delta Q_x, 0, Q_z)$ for m=$\pm$1 Skyrmion satellite peak, where $\delta Q_x \ll Q_z$. Here we assume that the titanium and lead atoms dominate x-ray diffraction signal, since the oxygen ions only contribute to a minor scattering intensity (<20% for bulk $PbTiO_3$) due to their small atomic scattering factor. $\mathbf{R}_{Pb/Ti,n}$ is the absolute position of the n-th unit cell Pb/Ti atom, which can be rewritten as $\mathbf{R}_{Pb/Ti,n} = \mathbf{R_0}_n + \mathbf{r}_{Pb/Ti,n}$. The first term $\mathbf{R_0}_n$ corresponds to the cell origin coordinates to the crystal origin. The last term designates the positions of the atoms relative to the cell origin. For convenience, we assume that the polarization change is attributed to the atomic position shifts in the Pb and Ti atoms. Here, the displacement of Pb and Ti from the center of the cell is identified as the source of the polarization, with the sign change in front of $b_{Pb/Ti}$ indicating that Pb and Ti move in opposite directions in their contribution to the polarization[2]:

$$r_{Pb,n} = -b_{Pb}\mathbf{P}_n \quad \text{(S2)}$$

$$r_{Ti,n} = \frac{1}{2}\mathbf{a_1} + \frac{1}{2}\mathbf{a_2} + \frac{1}{2}\mathbf{a_3} + b_{Ti}\mathbf{P}_n \quad \text{(S3)}$$

After plugging equations S2, S3 into S1, we can drop the term $e^{iQ\left(\frac{1}{2}\mathbf{a_1}+\frac{1}{2}\mathbf{a_2}+\frac{1}{2}\mathbf{a_3}\right)}$ as it is close to 1 at the Bragg condition. $b_{Pb/Ti}$ is estimated from the atomic displacement of Ti (0.3Å) and Pb (0.7 Å) and the spontaneous polarization in bulk $PbTiO_3$ (0.8 C/m^2) at 300 K. Then, we can rewrite the structure factor equation:

$$F(\mathbf{Q}) = \sum_n f_{Pb} e^{(-i\mathbf{Q}\cdot(\mathbf{R_0}_n - b_{Pb}\mathbf{P}_n))} + f_{Ti} e^{(-i\mathbf{Q}\cdot(\mathbf{R_0}_n + b_{Ti}\mathbf{P}_n))} \quad \text{(S4)}$$

The atomic form factor of Ti and Pb at 10 keV is estimated to be: $f_{Ti} = 22.4 + 1.2i$ and $f_{Pb} = 77.8 + 5.7i$ (e/atom) at the Bragg condition[3]. Here we consider $\mathbf{Q} = (\delta Q_x, 0, Q_z)$ to be at the Bragg condition with designated δQ_x and Q_z . By utilizing $e^{iQ_z\cdot R_{0z,n}} = 1$ and $b\mathbf{Q}\cdot\mathbf{P}_n \ll 1$, Eq. S4 becomes:

$$F((\delta Q_x, 0, Q_z)) = \sum_n [f_{Pb} e^{(-i(\delta Q_x R_{0\,x,n} - b_{Pb}\delta Q_x P_{x,n} - b_{Pb} Q_z P_{z,n}))} + f_{Ti} e^{(-i(\delta Q_x R_{0\,x,n} + b_{Ti}\delta Q_x P_{x,n} + b_{Ti} Q_z P_{z,n})))}] \approx \sum_n [e^{(-i\delta Q_x R_{0\,x,n})} (f_{pb}(1 + ib_{Pb}(\delta Q_x P_{x,n} + Q_z P_{z,n})) + f_{Ti}(1 - ib_{Ti}(\delta Q_x P_{x,n} + Q_z P_{z,n}))] \tag{S5}$$

We can then substitute the polarization obtained from the phase field calculation to $\mathbf{P}_n$ in S5. The diffraction intensity before pump excitation is:

$$I\big((\delta Q_x, 0, Q_z), t < t_0\big) \propto FF^* = \sum_{n,m} f_{Pb}^2 e^{-i\delta Q_x\left(R_{0\,x,n} - R_{0\,x,m}\right)} + \sum_{n,m} f_{Ti}^2 e^{-i\delta Q_x\left(R_{0\,x,n} - R_{0\,x,m}\right)} + \sum_{n,m} f_{pb} f_{Ti} \cos\left(\delta Q_x \left(R_{0_{x,n}} - R_{0_{x,m}}\right)\right) + \sum_{n,m} (f_{pb} + f_{Ti})(b_{Ti} f_{Ti} - b_{Pb} f_{Pb}) \sin\left(\delta Q_x \left(R_{0_{x,n}} - R_{0_{x,m}}\right)\right) (\delta Q_x P_{x,m} + Q_z P_{z,m}) + \sum_{n,m} (b_{Ti} f_{Ti} - b_{Pb} f_{Pb})^2 \cos\left(\delta Q_x \left(R_{0_{x,n}} - R_{0_{x,m}}\right)\right) (\delta Q_x P_{x,n} + Q_z P_{z,n})\big(\delta Q_x P_{x,m} + Q_z P_{z,m}\big) \tag{S6}$$

The diffraction intensity at the time delay of maximum phonon displacement (t_{max}) is:

$$I\big((\delta Q_x, 0, Q_z), t = t_{max}\big) \propto FF^* = \cdots + \sum_{n,m} (f_{pb} + f_{Ti})(b_{Ti} f_{Ti} - b_{Pb} f_{Pb}) \sin\left(\delta Q_x \left(R_{0_{x,n}} - R_{0_{x,m}}\right)\right) (\delta Q_x (P_{x,m} + \Delta P_{x,m}) + Q_z (P_{z,m} + \Delta P_{z,m})) + \sum_{n,m} (b_{Ti} f_{Ti} - b_{Pb} f_{Pb})^2 \cos\left(\delta Q_x \left(R_{0_{x,n}} - R_{0_{x,m}}\right)\right) (\delta Q_x (P_{x,n} + \Delta P_{x,n}) + Q_z (P_{z,n} + \Delta P_{z,n}))\big(\delta Q_x (P_{x,m} + \Delta P_{x,m}) + Q_z (P_{z,m} + \Delta P_{z,m})\big) \tag{S7}$$

The terms unrelated to polarizations represented by "…" are omitted, which are the same as those in equation S6. Here the ΔP corresponds to the change of polarization induced by the THz pulse. Furthermore, the dynamical response ΔI is absent at skyrmion SL peaks $(0,0,Q_z)$, which leads to $\sum_{n,m} (b_{Ti} f_{Ti} - b_{Pb} f_{Pb})^2 \big(2Q_z^2 \Delta P_{z,m} P_{z,n} + Q_z^2 \Delta P_{z,m} \Delta P_{z,n}\big) = 0$. We can then simplify the differential intensity difference between $t = t_{max}$ and $t < t_0$ as:

$$\Delta I \propto \sum_{n,m} (f_{pb} + f_{Ti})(b_{Ti} f_{Ti} - b_{Pb} f_{Pb}) \sin\left(\delta Q_x \left(R_{0_{x,n}} - R_{0_{x,m}}\right)\right) (\delta Q_x \Delta P_{x,m} + Q_z \Delta P_{z,m}) + \sum_{n,m} (b_{Ti} f_{Ti} - b_{Pb} f_{Pb})^2 \cos\left(\delta Q_x \left(R_{0_{x,n}} - \right.\right. \tag{S8}$$

$$R_{0_{x,m}}\Big)\Big)\left(\delta Q_x^2 \Delta P_{x,n}\Delta P_{x,m} + 2\delta Q_x^2 P_{x,n}\Delta P_{x,m} + 2\delta Q_x Q_z P_{x,n}\Delta P_{z,m} + 2\delta Q_x Q_z \Delta P_{x,n} P_{z,m} + 2\delta Q_x Q_z \Delta P_{x,n}\Delta P_{z,m}\right)$$

Then we can calculate the contribution of the remaining terms numerically in the polar skyrmion and polar vortex. The terms $2\delta Q_x Q_z P_{x,n}\Delta P_{z,m}$, $2\delta Q_x Q_z \Delta P_{x,n} P_{z,m}$, $2\delta Q_x Q_z \Delta P_{x,n}\Delta P_{z,m}$ would flip sign between the $+Q_x$ and $-Q_x$ satellites, therefore, they are antisymmetric terms for Q_x. In a polar skyrmion, the $2\delta Q_x Q_z \Delta P_{x,n} P_{z,m}$ term dominates the dynamical response. On the other hand, in the polar vortex structures, the symmetric term $Q_z^2 \Delta P_{z,n} P_{z,m}$ dominates the dynamical response, which is consistent with the experimental observation that the dynamical signals in $+Q_x$ and $-Q_x$ are in phase[4]. Noted the Q_z^2 term in vortex does not vanish as the dynamical response at SL peak is presented.

The above analysis can also explain the out-of-phase oscillation between *m*=1 and -1 peaks, as well as between *m*=2 and -2 peaks. To explain the opposite phases between *m*=1 and 2 peaks, and between *m*=-1 and -2 peaks, we examined the contribution to first and second order satellite intensity. Phase-field calculation analyzed the contribution to diffraction intensity from domain inside the bubbles (c+), matrix between the bubbles (c-) and the skyrmion domain wall (DW). We found that the first order satellite intensity is mainly contributed by c+ and c-, while the second order intensity is mainly contributed by DW (Fig. 3f-h, Fig. S3). We then calculated the contribution to diffraction intensity changes from c+, c- and DW based on Eq. S8. The analytical results confirm that the first and second order satellite intensity oscillate out of phase (Fig. 3h), which is quantitatively agree with the phase field diffraction simulation results. The same sign changes in dynamical diffraction intensity from c+, c- and DW contributions (Fig. S3).

<u>Supplementary Text 2: Calibration between computational and experimental results</u>

In this session, we quantitatively assess the atomic motion by comparing the computational and experimental results. We use the atomic positions and displacements of Pb, Sr, Ti and O from the atomistic model to calculate the diffraction intensity (Fig. S10a). To match the experimentally observed intensity changes, the eigen displacement as obtained from the atomistic modeling needs to be multiplied by a factor of 60. As a result, the calibrated Pb displacement and polarization change in mode A and mode B can be obtained (Fig. S10c-f). We estimate the mean value of Pb displacement is ~2 pm. The corresponding shear strain was calculated based on this scaling.

	$PbTiO_3$	$SrTiO_3$
Landau coefficients		
a_1 (m^2 N C^{-2})	3.8×10^5 (T-752)	4.05×10^7 [1/tanh(54/T)-1.056]
a_{11} (m^6 N C^{-4})	-7.3×10^7	1.7×10^9
a_{12} (m^6 N C^{-4})	7.5×10^8	3.9×10^9
a_{111} (m^{10} N C^{-6})	2.6×10^8	0
a_{112} (m^{10} N C^{-6})	6.1×10^8	0
a_{123} (m^{10} N C^{-6})	-3.7×10^9	0
Electrostrictive coefficients		
Q_{11} (m^4 C^{-2})	0.089	0.0457
Q_{12} (m^4 C^{-2})	-0.026	-0.0135
Q_{44} (m^4 C^{-2})	0.03375	0.0096
Elastic stiffness tensor		
C_{11}(N m^{-2})	1.76×10^{11}	1.76×10^{11}
C_{12}(N m^{-2})	7.937×10^{10}	7.937×10^{10}
C_{44}(N m^{-2})	1.111×10^{11}	1.111×10^{11}
Mass coefficient of polarization		
μ (J m A^{-2})	7.5×10^{-17}	7.5×10^{-17}
Damping coefficient of polarization		
γ (J m A^{-2})	2×10^{-7}	2×10^{-7}
Mass density		
ρ (kg m^{-3})	7.5×10^3	7.5×10^3
Elastic stiffness damping coefficient		
β (s)	6×10^{-12}	6×10^{-12}
Polarization-atom position constant		
b_{Pb}/b_{Sr} (C^{-1} m^3)	1.01×10^{-11}	1.01×10^{-11}
b_{Ti} (C^{-1} m^3)	-0.56×10^{-11}	-0.56×10^{-11}
b_{O_I} (C^{-1} m^3)	-3.66×10^{-11}	-3.66×10^{-11}
$b_{O_{II}}$ (C^{-1} m^3)	-3.15×10^{-11}	-3.15×10^{-11}
Lattice mismatch		
ε_{11}	0.11%	0
ε_{22}	0.26%	0

Table S1: Parameters for dynamic phase field simulations and diffraction pattern calculation.

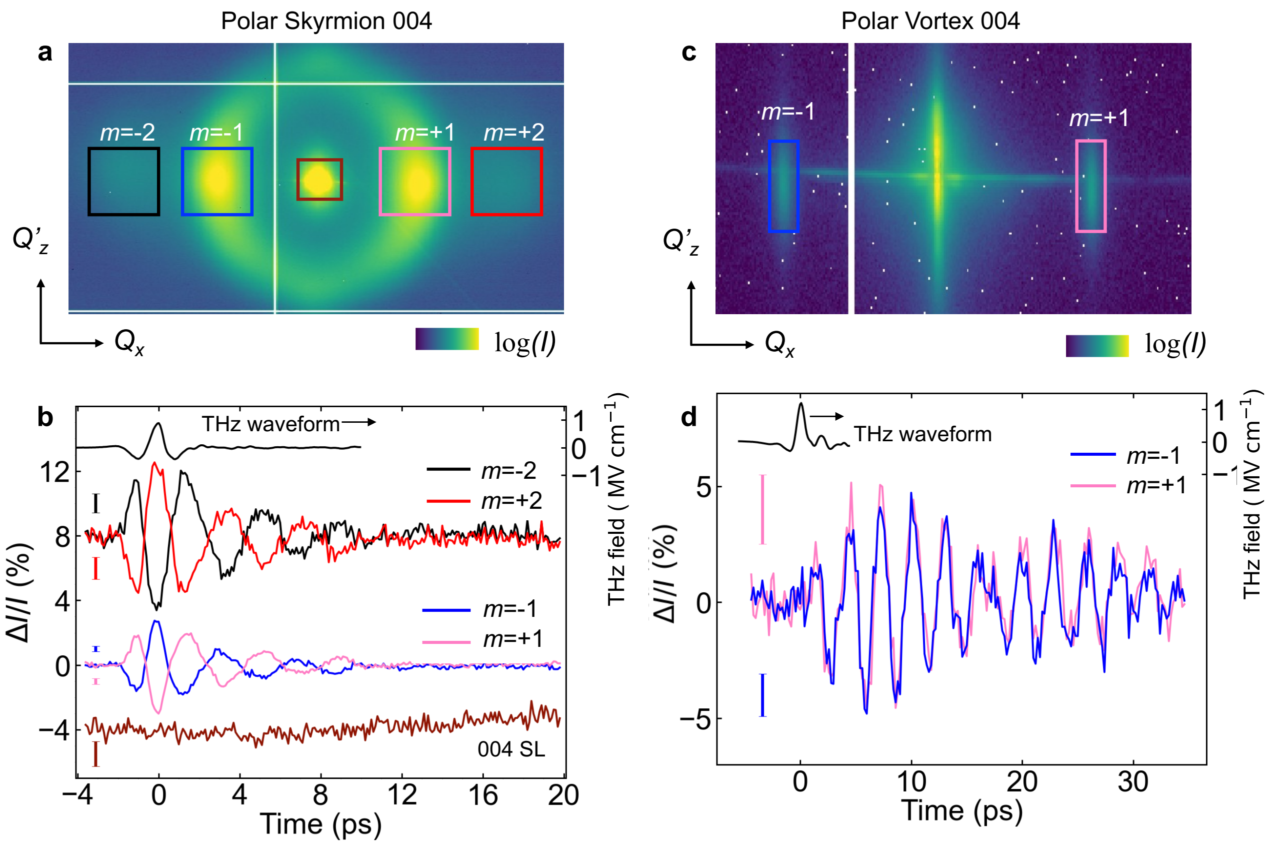

Fig. S1. Dynamics of polar skyrmion and vortices near 004 Bragg peak. a Detector image near 004 Bragg peak of polar skyrmion at 220 K. **b** Normalized intensity evolution ($\Delta I/I$) as a function of time, along with the m=±1, ±2 orders of the skyrmion satellite peaks, and SL peak intensity with the region of interest (ROI) choice highlighted by the colored box in **a**. **c** Detector image near 004 Bragg peak of polar vortices at 300 K. **d** Normalized intensity evolution ($\Delta I/I$) of the m=±1 order polar vortex satellite peaks as a function of time. The colored box also highlights the ROI choice in **c**. The error bar represents the standard deviation of the data points before time zero.

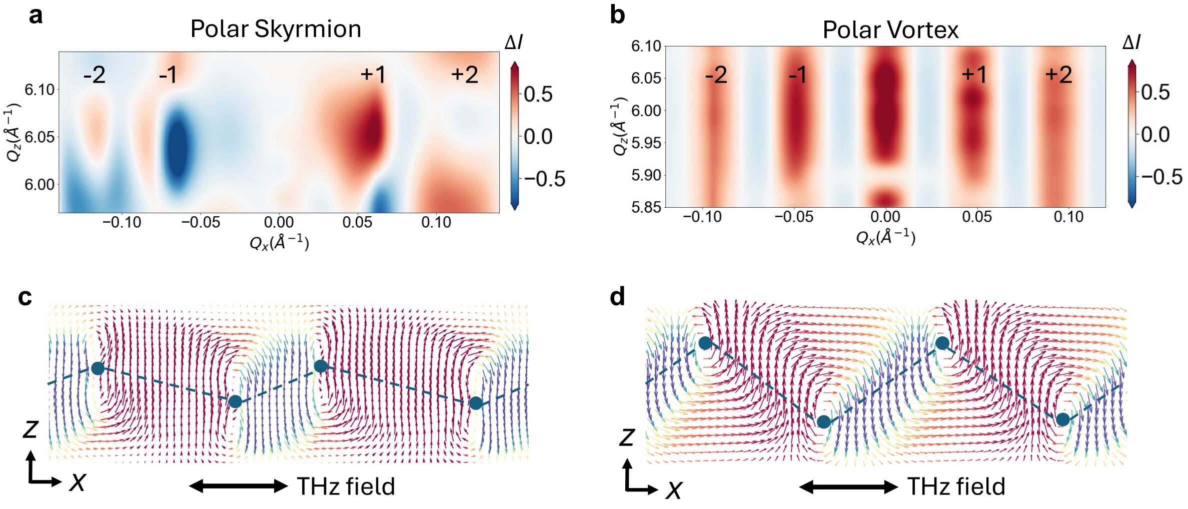

Fig. S2. Dynamical phase-field simulation of THz-driven polar skyrmions and polar vortices. Simulated diffraction intensity modulation (**Δ***I*) from THz field at the delay of maximum phonon displacement for (**a**) polar skyrmions and (**b**) polar vortices, based on the snap shot of the dynamical polarization configuration shown in (**c**, **d**), respectively. The distorted polarization structure of (**c**) polar skyrmion shows uneven tilt angles of domain wall core in *c*+ and *c*- domains while that of (**d**) polar vortex shows even tilt angles.

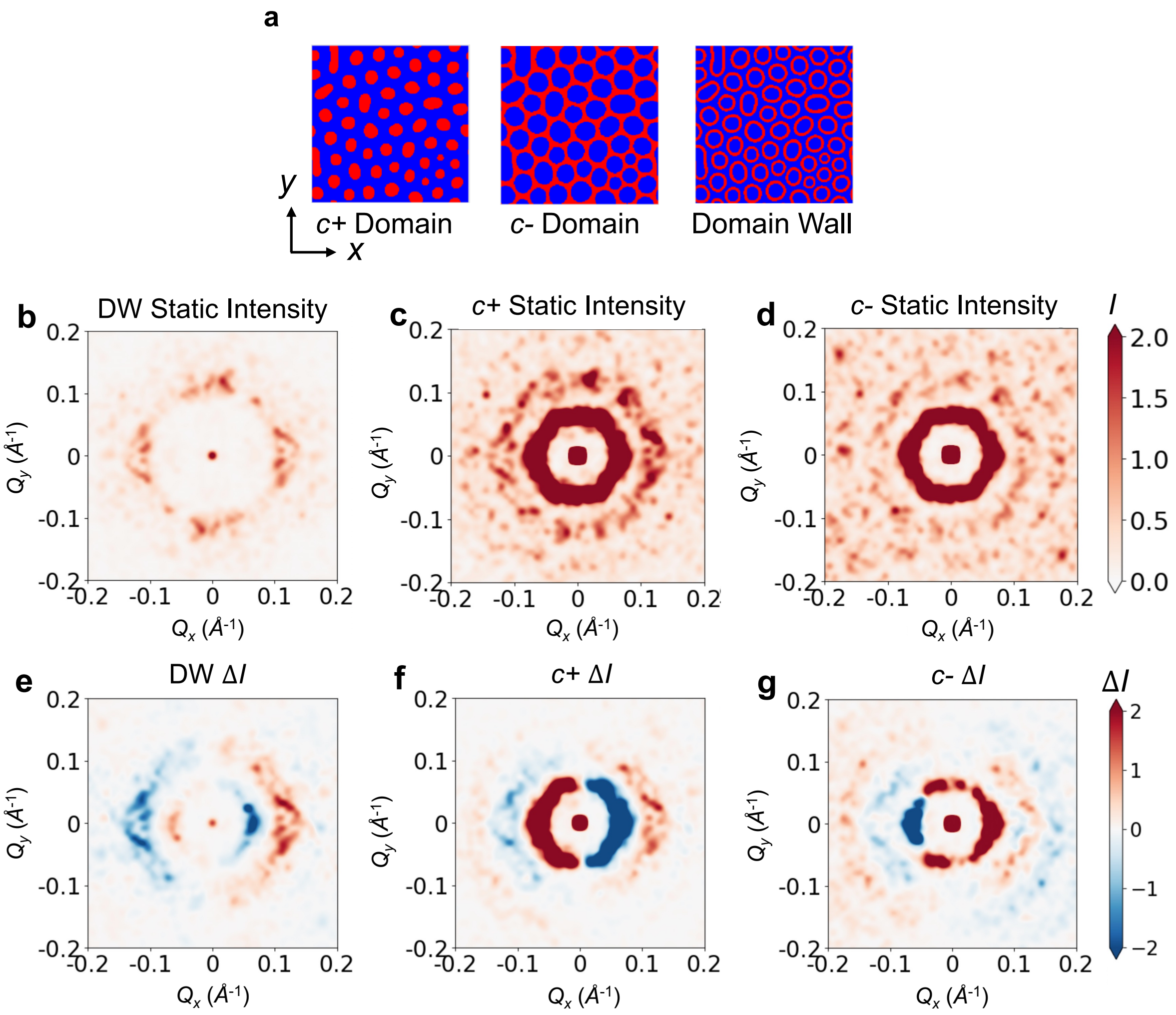

Fig. S3. Simulations of the static and dynamic diffraction intensity from skyrmion domains and domain walls. a Phase-field simulation of diffraction intensity of polar skyrmion. The structure factor of the Domain inside the skyrmion bubbles (*c*+), the matrix between the bubbles (*c*-), and the skyrmion domain wall (DW) are selectively extracted (selected areas as highlighted in red) by setting the atomic factor outside the selected phase to zero (the areas with zero atomic factor are highlighted in blue). Phase-field simulation of contribution to static diffraction intensity from (**b**) DW, (**c**) *c*+ and (**d**) *c*- component of polar skyrmions. The diffraction intensity modulation at 1/4 of the 0.3THz phonon periodicity has been itemized into contribution from (**e**) DW, (**f**) *c*+ and (**g**) *c*- component of polar skyrmions. Note the analytical approximation results qualitatively agrees with the phase field results.

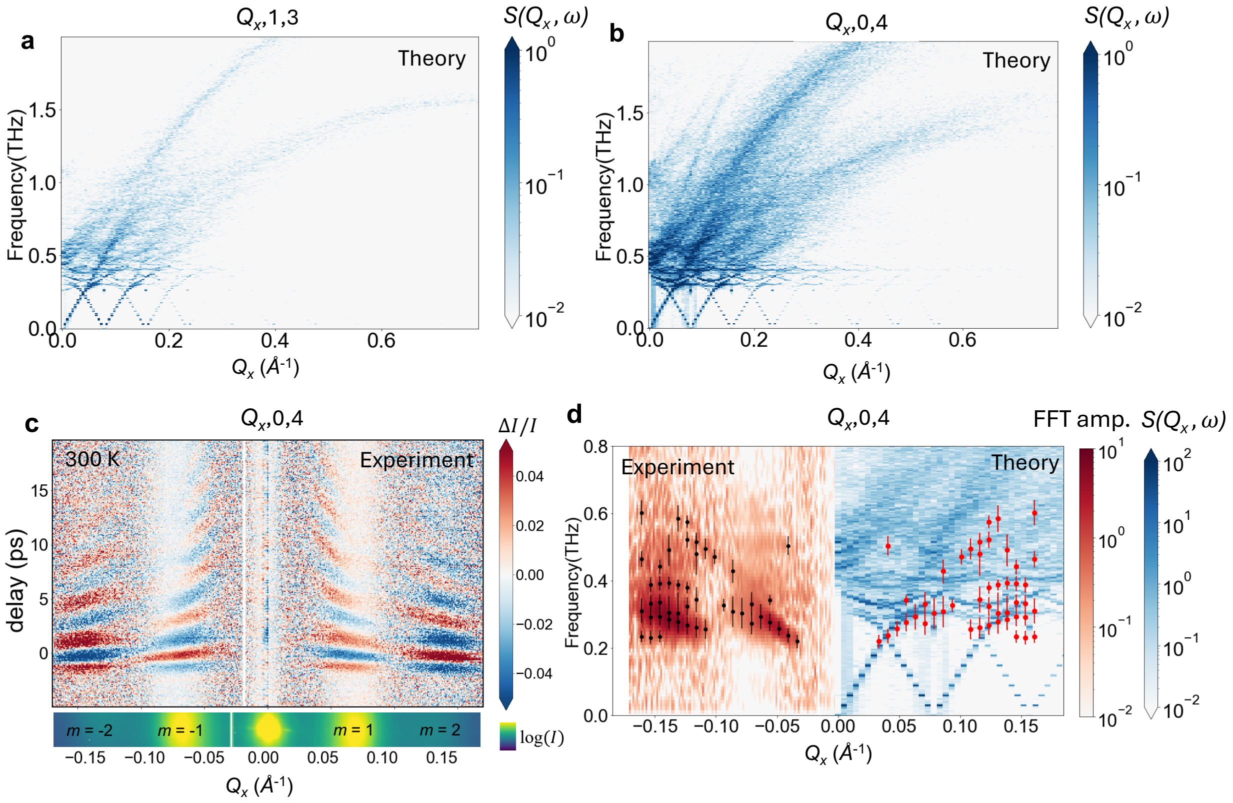

Fig. S4. Phonon dispersion calculated by the atomistic model and compared with experimental results. Phonon dispersion calculated for (**a**) (Q_x,1,3) and (**b**) (Q_x,0,4). The results are from the molecular dynamics calculation of 16 skyrmions Despite different Bragg peaks are probed, the time-domain responses and dispersions are similar, representing the generality of the modified phonon dispersion. Each skyrmion is composed of 20 by 20 by 24 unit cells with 16 layers of $PbTiO_3$ and 8 layers of $SrTiO_3$ unit cells. The THz pump XRD probe of satellite peaks around (0,0,4) Bragg peak has been measured experimentally and plotted in **c**. **d** Fourier spectra of the time evolution of the relative intensity change shown in **c**, compared with atomistic simulation results. The color map of the simulation represents the amplitude of the dynamic structure factor $S(Q_x, \omega)$. The red and black dots with error bars showing the FWHM of the Lorentzian peak fitting of the experimental Fourier spectra at each Q_x.

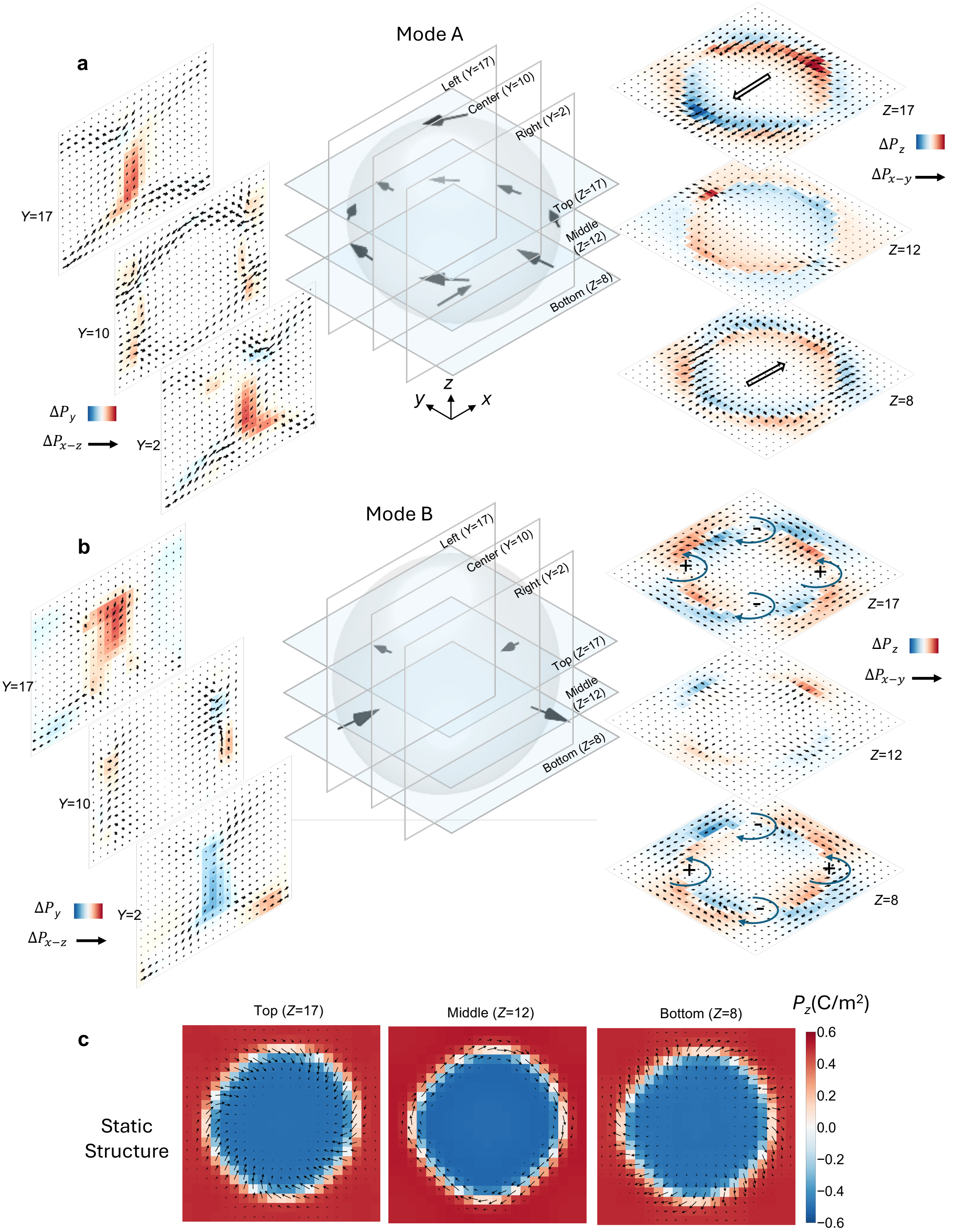

Fig. S5. Polarization changes at the peak of their motions calculated by the atomistic model. The *x*-*y* and *x*-*z* cuts of the polarization changes induced by mode A (**a**) and B (**b**). **c** *x*-*y* cuts of static polarization in polar skyrmion. The 20 by 20 by 24 supercell index is used as guidance for plane cuts. *Z* is the out-of-plane unit cell index. *Z*=0-3, and 20-23 are $SrTiO_3$ unit cells and *Z*=4-19 are $PbTiO_3$ unit cells.

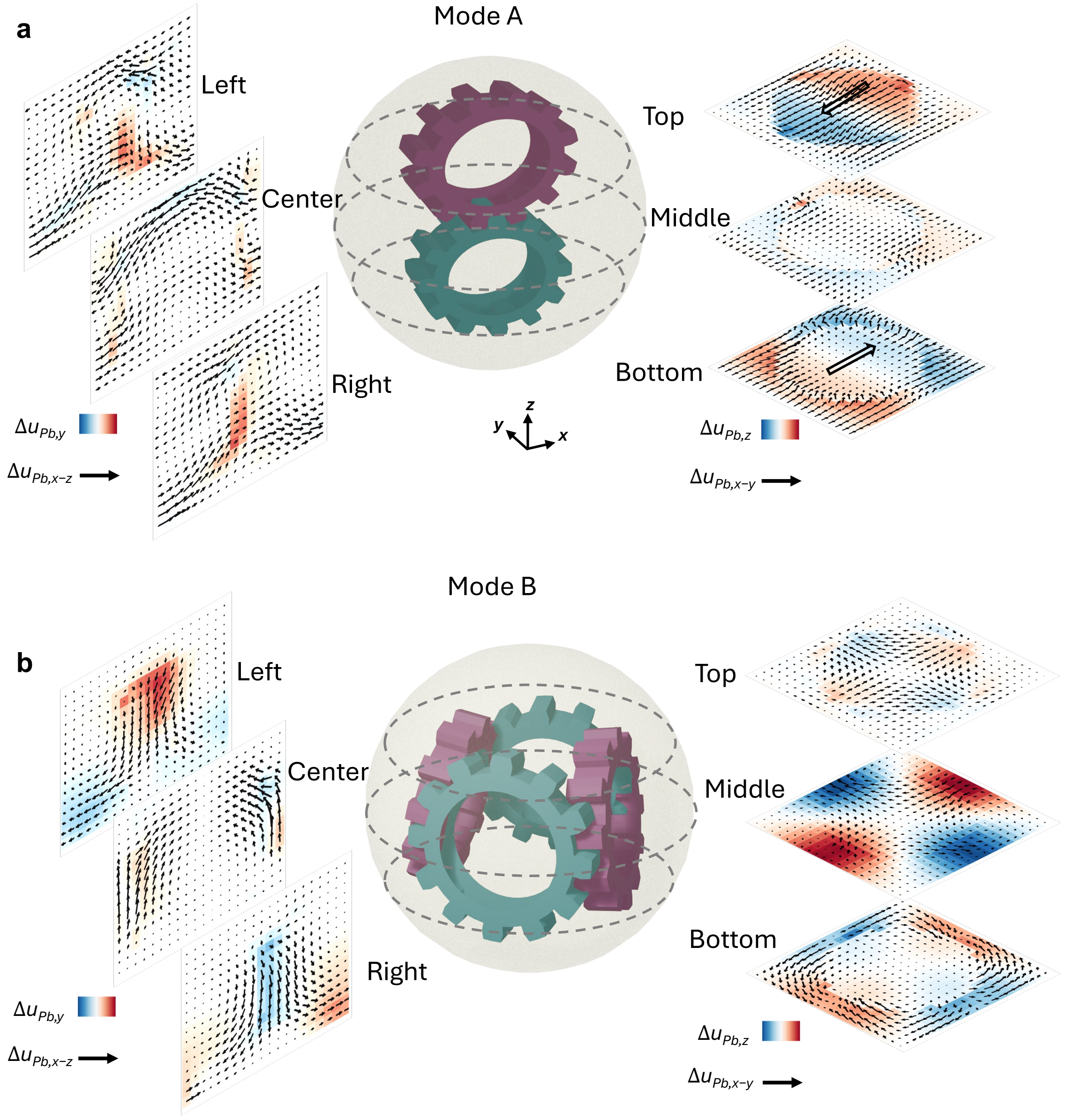

Fig. S6. Pb displacements at the peak of their motions calculated by the atomistic model. The *x*-*y* and *x*-*z* cuts of the lead displacement induced by mode A (**a**) and B (**b**).

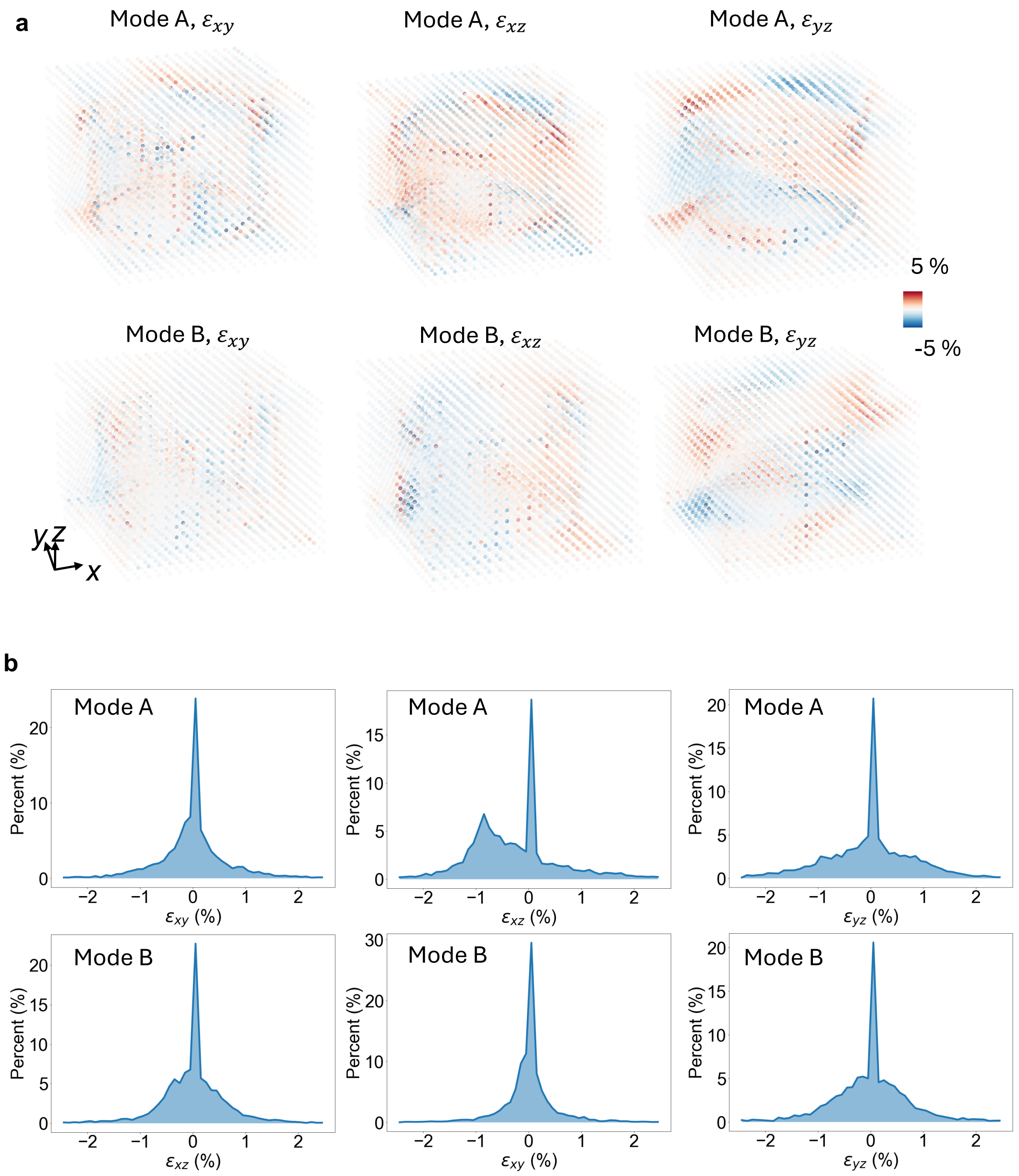

Fig. S7. Shear strain induced by mode A and B. a Shear strain map of mode A and B. Each dot represents the strain value shown in the color bar. **b** Distribution of shear strain in each $PbTiO_3$ cell inside the polar skyrmion from mode A and B.

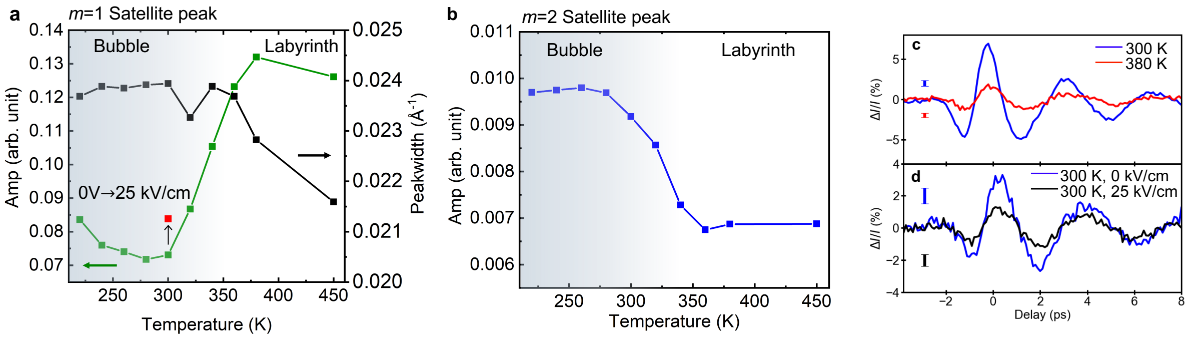

Fig. S8. Temperature and field dependence of the dynamical and static responses in polar skyrmions. a Experimental results of the static first-order satellite peak intensity and peak-width evolution as a function of temperature. The in-plane DC electrical bias induced peak intensity change at 300 K is highlighted by a black arrow between the unbiased green data point and the biased red data point. **b** Experimental results of the static second-order satellite peak intensity evolution as a function of temperature. The normalized integrated intensity evolution ($\Delta I/I$) at the first order of polar skyrmion near 004 peak induced by an intense THz pulse at (**b**) 300 K and 380 K, and (**c**) at 0 kV/cm and 20 kV/cm in-plane electric field. The intensity change is integrated in a similar way as shown in Fig. S1. to enhance the signal to noise ratio, we sum the signal in the *m*=+1 and *m*=-1 satellite peaks as $(\Delta I_{m=1} - \Delta I_{m=-1})/(I_{m=1} + I_{m=-1})$. The error bar represents the standard deviation of the data points before time zero data.

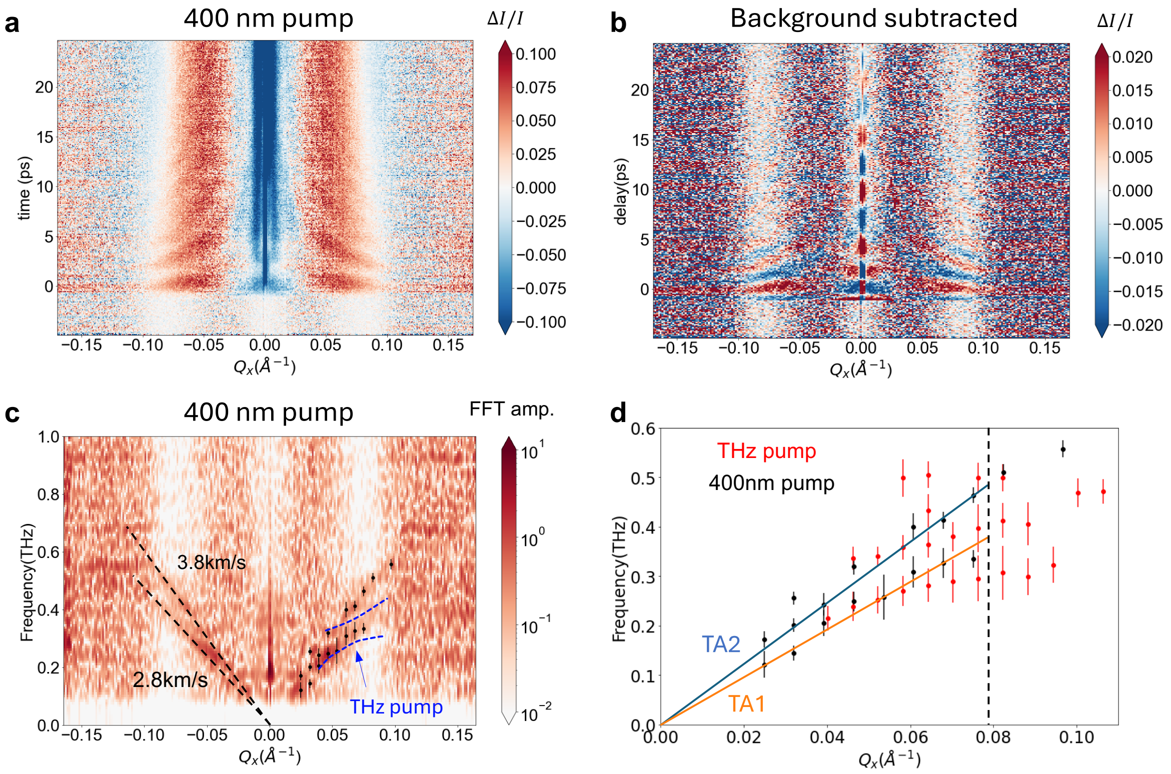

Fig. S9. Dynamical response of the polar skyrmions to 400 nm optical pump at 300 K. a Dynamical response of the relative X-ray diffraction intensity changes near 004 peak normalized to the static diffraction intensity before time zero is plotted as a function of delay and Q_x. **b** Exponential decay background is fitted and subtracted from the data. The remaining oscillatory signal is plotted. **c** Fourier spectra of the background subtracted dynamical responses are plotted as a function of frequency and Q_x. The linear dispersion originating from TA and LA branches are highlighted by dashed lines and their speeds of sound are annotated. The THz pump result is shown by dashed blue lines. **d** Extracted dispersion data from 400 nm pump (black) and THz pump (red) are plotted side-by-side overlaid with the TA1 and TA2 branch dispersions, which slopes are consistent with the transverse speeds of sound of $PbTiO_3$ along the [100] directions[5]. The error bars represent the widths of the Lorentzian peak fittings. The vertical dashed line indicates the Q_x position of first satellite peak (m = 1). The error bars show the FWHM of the Lorentzian peak fitting of the experimental Fourier spectra at each Q_x.

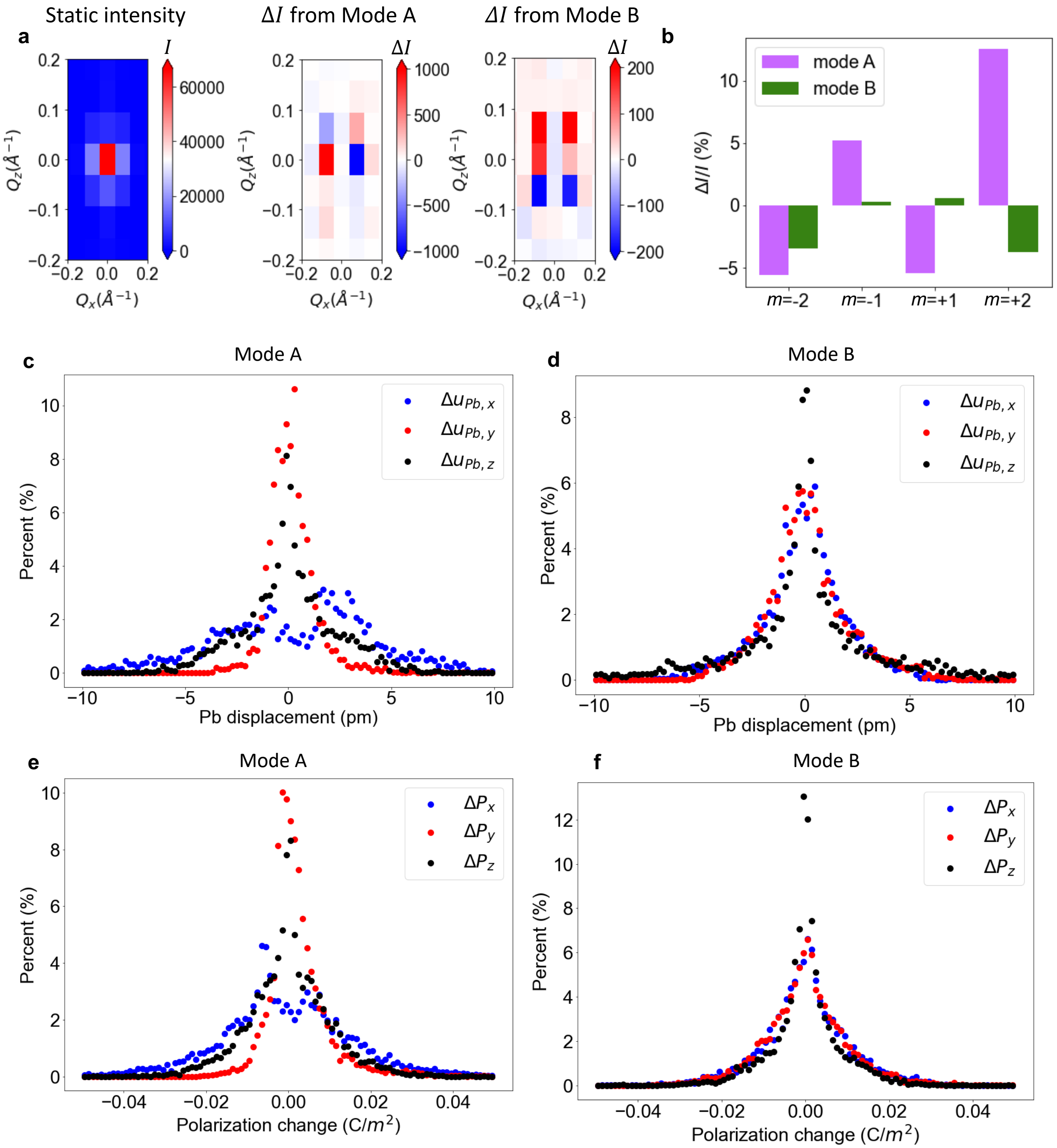

Fig. S10. Calibrate atomistic results to experimental observations. **a** Diffraction simulation with atomistic simulation inputs. From left to right: static diffraction intensity, ΔI from mode A, and ΔI from mode B. **b** Induced $\Delta I/I_0$ signal from mode A and B. After normalizing the dynamical response by calibrating with experimental results, we plot the distribution of Pb displacement (**c,d**) and the polarization change (**e,f**) of mode A and B.

References

1. Yang, T. et al. Computing diffraction patterns of microstructures from phase-field simulations. Acta Materialia **239**, 118258 (2022).
2. Meyer, B., & Vanderbilt, D. Ab initio study of ferroelectric domain walls in $PbTiO_3$. Physical Review B **65** 104111 (2002).
3. Chantler, C. T. Theoretical form factor, attenuation, and scattering tabulation for z= 1–92 from e= 1–10 eV to e=0.4–1.0 meV. Journal of Physical and Chemical Reference Data **24**, 71–643 (1995).
4. Li, Q. et al. Subterahertz collective dynamics of polar vortices. Nature **592**, 376–380 (2021).
5. Kalinichev, A., Bass, J., Sun, B. & Payne, D. Elastic properties of tetragonal $PbTiO_3$ single crystals by Brillouin scattering. Journal of materials research **12**, 2623–2627 (1997).
