# Supplementary material for: Terahertz-field activation of polar skyrons
Source: Nat Commun. 2025 Oct 9;16:8994. doi: 10.1038/s41467-025-64033-6 (PMC12511383; doi:10.1038/s41467-025-64033-6)
Supplement: Supplementary file 2 — Description Of Additional Supplementary File [file 41467_2025_64033_MOESM2_ESM.pdf]

Description of Additional supplementary files

Supplementary Movie 1. Movie of polarization change vectors with color map of polarization change along x (left) and polarization change along z (right) produced by mode A.

Supplementary Movie 2. Movie of lead displacement vectors with color map of lead displacement along x (left) and lead displacement along z (right) produced by mode A.

Supplementary Movie 3. Movie of polarization change vectors with color map of polarization change along x (left) and polarization change along z (right) produced by mode B.

Supplementary Movie 4. Movie of lead displacement vectors with color map of lead displacement along x (left) and lead displacement along z (right) produced by mode B.
